# Prognostic Implications of T Cell Receptor Repertoire Diversity in Cervical Lymph Nodes of Oral Squamous Cell Carcinoma Patients

**DOI:** 10.3390/ijms26157073

**Published:** 2025-07-23

**Authors:** Kenichi Kumagai, Yoshiki Hamada, Akihisa Horie, Yudai Shimizu, Yoshihiro Ohashi, Reo Aoki, Taiki Suzuki, Koji Kawaguchi, Akihiro Kuroda, Takahiro Tsujikawa, Kazuto Hoshi, Kazuhiro Kakimi

**Affiliations:** 1 Department of Oral-Maxillofacial Surgery and Orthodontics, The University of Tokyo Hospital, Tokyo, 7-3-1 Hongo, Bunkyo-ku, Tokyo 113-8655, Japan; kumagaik-ora@h.u-tokyo.ac.jp (K.K.); hoshi-ora@h.u-tokyo.ac.jp (K.H.); 2 Department of Oral and Maxillofacial Surgery, School of Dental Medicine, Tsurumi University, 2-1-3 Tsurumi, Tsurumi-ku, Yokohama 230-8501, Japan; hamada-y@tsurumi-u.ac.jp (Y.H.); pd25005@stu.tsurumi-u.ac.jp (Y.S.); kawaguchi-k@tsurumi-u.ac.jp (K.K.); 3 Department of Oral and Maxillofacial Surgery, Kanto Rosai Hospital, 1-1 Kizukisumiyoshicho, Kawasaki 211-8510, Japan; horiea@kantoh.johas.go.jp (A.H.); y.ohashi@kantoh.johas.go.jp (Y.O.); r.aoki.oms@gmail.com (R.A.); suzukitaiki000@gmail.com (T.S.); 4 Department of Gastrointestinal Surgery, Graduate School of Medicine, The University of Tokyo, 7-3-1 Hongo, Bunkyo-ku, Tokyo 113-8655, Japan; akihiro.kuroda.0430@gmail.com; 5 Department of Otolaryngology-Head and Neck Surgery, Kyoto Prefectural University of Medicine, Kyoto, 465 Kajiicho, Kamigyo-ku, Kyoto 602-8566, Japan; tu-ji@koto.kpu-m.ac.jp; 6 Department of Immunology, Kindai University Faculty of Medicine, 377-2 Ohno-Higashi, Osaka-Sayama, Osaka 589-8511, Japan

**Keywords:** oral squamous cell carcinoma (OSCC), tumor-draining lymph node (TDLN), T cell receptor (TCR) repertoire, lymph node metastasis, prognostic biomarker, Shannon diversity index, risk stratification

## Abstract

The immune landscape of tumor-draining lymph nodes (TDLNs) plays a critical role in shaping antitumor responses and influencing prognosis in oral squamous cell carcinoma (OSCC). Among patients with lymph node (LN) metastasis, clinical outcomes vary widely, yet reliable biomarkers for prognostic stratification remain limited. This study aimed to identify immune features in tumors and LNs that differentiate between favorable and poor outcomes in OSCC patients with nodal metastasis. We analyzed T cell receptor (TCR) CDR3 repertoires and the expression of immune-related genes in primary tumors and paired sentinel LNs from OSCC patients who underwent tumor resection and lymphadenectomy. Patients were divided into three groups: Group A (no nodal metastasis), Group B1 (metastasis without recurrence), and Group B2 (metastasis with recurrence). TCR diversity was assessed using the Shannon index. The expression of immune-related genes (e.g., *CD3E*, *CD4*, *CD8B*, *FOXP3*, *CTLA4*, *IL2*, *IL4*) was measured by quantitative PCR and normalized to *GAPDH*. TCR diversity was lower in tumors than in non-metastatic LNs, reflecting clonal expansion. Metastatic LNs exhibited tumor-like diversity, suggesting infiltration by tumor-reactive clones. Tumor gene expression did not differ across groups, but LNs from metastatic cases showed the reduced expression of several immune genes. Notably, *CD3E*, *CD8B*, *CTLA4*, *IL2*, and *IL4* distinguished B1 from B2. The immune profiling of LNs offers superior prognostic value over tumor analysis in OSCC patients with LN metastasis. LN-based evaluation may aid in postoperative risk stratification and personalized postoperative management and could inform decisions regarding adjuvant therapy and follow-up strategies.

## 1. Introduction

Oral squamous cell carcinoma (OSCC) is the eleventh most common malignancy worldwide and remains a major public health concern [1]. Although multidisciplinary therapeutic strategies, including surgery, radiation, and chemotherapy, have advanced, the prognosis for patients with OSCC is still suboptimal, primarily due to loco-regional recurrence and distant metastasis [2]. In particular, the presence of cervical lymph node (LN) metastasis at the time of surgery is a well-established indicator of poor prognosis [3,4]. However, even among patients with LN involvement, clinical outcomes are heterogeneous: some experience early postoperative recurrence or distant metastasis, while others maintain long-term remission with no signs of disease progression.

Current histopathological and molecular markers fail to reliably distinguish high-risk individuals who require intensive adjuvant treatment from those who may be managed with careful surveillance [5]. Although features such as extranodal extension (ENE), tumor budding, and depth of invasion have been used for prognostic stratification, they often fail to capture the immunological status of tumor-draining lymph nodes and do not adequately predict recurrence risk in all cases. This lack of predictive tools highlights an urgent need to identify novel prognostic indicators that can stratify patients with LN metastasis more accurately. Cervical draining LNs (CDLNs) play a dual role in OSCC. On the one hand, they act as key sites for initiating antitumor immune responses, where tumor-associated antigens are presented to T cells by antigen-presenting cells (APCs), facilitating the activation of tumor-reactive lymphocytes [6]. On the other hand, CDLNs also serve as conduits for tumor cell dissemination, providing a niche for metastatic colonization and immune evasion [7]. These opposing functions complicate the immunological interpretation of LN status and its clinical implications [8,9].

T cell receptor (TCR) repertoire analysis enables a high-resolution view of T cell diversity and clonal dynamics, offering insights into immune activation, clonal expansion, and potential tumor reactivity [10,11]. By analyzing TCR repertoires in both primary tumors and paired CDLNs, this study sought to identify immunological patterns—such as clonal expansion, restricted diversity, and potential T cell dysfunction—that reflect the quality of antitumor immunity and correlate with clinical outcomes.

In this study, we aimed to explore predictive immune correlates in OSCC patients with LN metastasis by characterizing the TCR repertoire of primary tumors and corresponding CDLNs. Our goal was to identify immune-based biomarkers that can distinguish patients at high risk of recurrence from those with favorable prognosis, ultimately contributing to improved risk stratification and the development of TCR-guided personalized immunotherapy strategies.

## 2. Results

### 2.1. Patient Grouping and Survival Outcome

Sixteen patients with oral squamous cell carcinoma (OSCC; clinical stage II–IVB) who underwent primary tumor resection and neck dissection were included in this study. The demographic and clinical characteristics of these patients are summarized in Table 1. Based on the presence or absence of LN metastasis, patients were classified into two main groups: Group A (*n* = 5), with no LN metastasis, and Group B (*n* = 11), with pathologically confirmed nodal involvement.

Among the 11 patients in Group B, further subclassification was performed according to a postoperative clinical course. Six patients who showed no evidence of disease recurrence during the follow-up period were assigned to Group B1, while the remaining five patients, who developed local recurrence or distant metastasis, were classified as Group B2.

**Figure 1:**
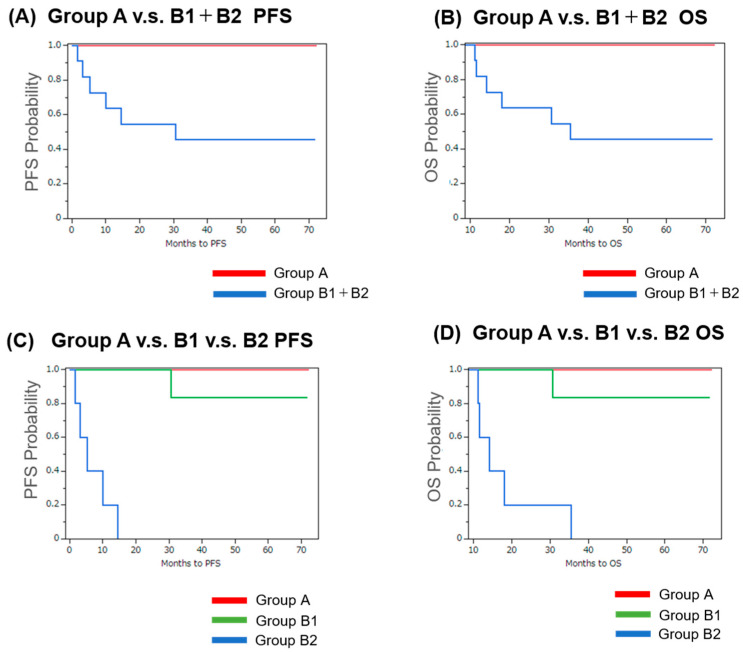
Kaplan–Meier analysis of progression-free survival (PFS) and overall survival (OS) in OSCC patients. Kaplan–Meier curves illustrating progression-free survival (PFS) and overall survival (OS) among oral squamous cell carcinoma (OSCC) patients stratified by LN status and clinical outcome. (**A**,**B**) Comparison between Group A (patients without LN metastasis, *n* = 5) and Group B (patients with nodal metastasis, *n* = 11; B1 + B2 combined) for PFS (**A**) and OS (**B**). (**C**,**D**) Comparison among Group A, Group B1 (nodal metastasis without recurrence, *n* = 6), and Group B2 (nodal metastasis with recurrence or distant metastasis, *n* = 5) for PFS (**C**) and OS (**D**). Statistical analysis was performed using the log-rank test. A *p*-value of <0.05 was considered statistically significant.

As previously reported [12,13,14], the patients in Group B (with LN metastasis) had significantly worse progression-free survival (PFS) and overall survival (OS) compared to those in Group A (without nodal involvement), as evaluated by Kaplan–Meier analysis (Figure 1A,B). Furthermore, within the LN-positive cohort, the patients in Group B2 exhibited significantly shorter PFS—as expected—but also markedly reduced OS compared to Group B1, indicating a poor prognosis for patients with postoperative recurrence or metastasis (Figure 1C,D).

### 2.2. Investigation of Histopathological Prognostic Factors in OSCC Patients

We compared the clinical stage, Y-K classification [14,15] of the primary tumor, and histopathological characteristics of metastatic LNs between Group B1 and Group B2 (Table 1 and Table 2). However, none of these parameters effectively discriminated between patients who experienced postoperative recurrence and those who did not. Notably, both focal and invasive/cystic patterns of LN metastasis were observed in patients from both groups (Supplementary Figure S1). Recently, an international expert consensus was established to standardize the pathological diagnosis and reporting of extranodal extension (ENE) in head and neck cancers [16]. This consensus has clarified the diagnostic criteria for ENE, including the subclassification into minor and major ENE, which has been reported to correlate with clinical outcomes. In general, the absence of ENE is associated with favorable prognosis, whereas the presence of major ENE is considered an indicator of poor prognosis. However, in our analysis, some patients in Group B1 (favorable prognosis) exhibited major ENE, while ENE-negative cases were also observed in Group B2 (poor prognosis). These findings suggest that ENE status alone may not be sufficient to stratify the risk of recurrence or predict clinical outcomes accurately in this cohort. This apparent discrepancy with previous reports may reflect cohort size, retrospective design, or other confounding factors, and it is discussed in further detail below. Therefore, a more comprehensive evaluation that incorporates immunological factors may be necessary for precise prognostication.

### 2.3. TCR Repertoire Diversity Reflects Nodal Metastasis and Tumor-Specific Clonal Expansion

**Figure 2:**
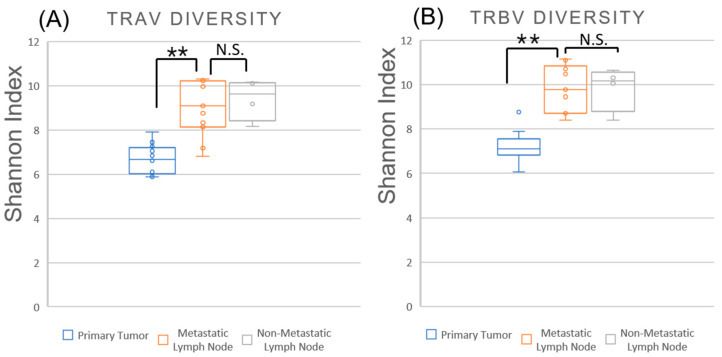
T cell receptor (TCR) diversity in primary tumors and cervical LNs in OSCC patients. Scatter plots showing Shannon diversity indices for TCR α (TRA) and β (TRB) chain repertoires in primary tumors (blue), metastatic LNs (orange), and non-metastatic LNs (gray) from patients with oral squamous cell carcinoma (OSCC). Statistical comparisons were performed using the Mann–Whitney U test. Significance was indicated as follows: ** *p* < 0.01; N.S., not significant.

To elucidate the expansion and distribution of T cell clones in primary tumors and their corresponding sentinel LNs in OSCC patients, we analyzed the TCR CDR3 sequences in tumor tissues and paired sentinel LNs. The diversity and evenness of the TCR repertoire were quantified using the Shannon diversity index, which captures both the number of unique clonotypes and their relative abundances (Figure 2 and Supplementary Table S1). First, we compared the diversity of the TCR α chain (TRA) and TCR β chain (TRB) repertoires between primary tumors and level IB LNs. The diversity index values for TRA in primary tumors, metastatic LN, and non-metastatic LN were 6.66 ± 0.65, 9.27 ± 1.27, and 9.16 ± 0.94, respectively. Similarly, those for TRB in primary tumors, metastatic LN, and non-metastatic LN were 7.24 ± 0.62, 9.83 ± 1.07, and 9.85 ± 1.01, respectively.

Consistent with previous reports [10,11], both metastatic and non-metastatic LNs exhibited higher TCR diversity than primary tumors for both TRA and TRB repertoires, reflecting the inherently diverse TCR repertoire of lymphoid tissues. Although the difference in diversity between metastatic and non-metastatic LNs did not reach statistical significance, metastatic nodes tended to show lower Shannon indices, and in several cases, the values were comparable to those observed in tumors. This trend may suggest the enrichment of tumor-associated T cell clones in metastatic LNs; however, this interpretation remains speculative in the absence of direct evidence from TCR overlap or clonotype tracking. The reduced TCR diversity in primary tumors is consistent with clonal expansion, possibly reflecting the selective proliferation of tumor-reactive T cells within the tumor microenvironment. Together, these observations point to potential immunological differences between LNs with and without metastasis (Group B vs. Group A), providing a rationale for further comparison between subgroups B1 and B2.

### 2.4. Restricted TCR Diversity in LNs Correlates with Favorable Prognosis

**Figure 3:**
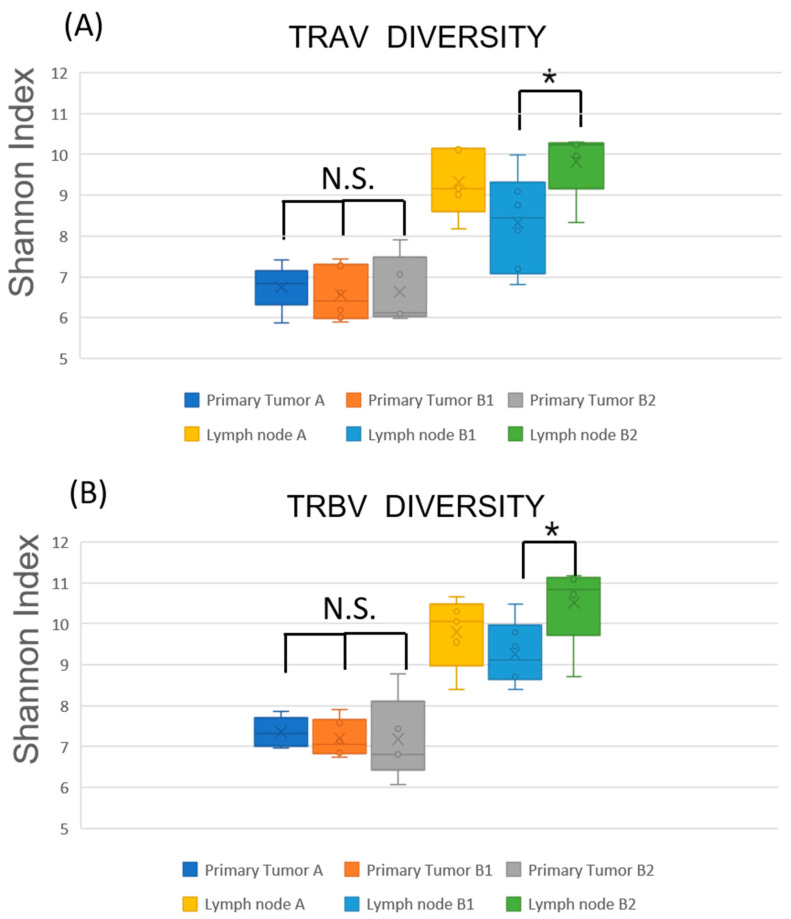
Comparison of TCR diversity across patient subgroups with different prognoses in OSCC. Box plots and scatter plots showing the Shannon diversity indices for TCR α (TRA) and β (TRB) chain repertoires in primary tumors and cervical LNs from OSCC patients, categorized into Group A (no LN metastasis), Group B1 (nodal metastasis without recurrence), and Group B2 (nodal metastasis with recurrence). Color coding: blue box, primary tumor (Group A); orange, primary tumor (Group B1); gray, primary tumor (Group B2); yellow, LN (Group A); light blue, LN (Group B1); green, LN (Group B2). Statistical comparisons were performed using the Mann–Whitney U test. * *p* < 0.05; N.S., not significant.

To compare the antitumor T cell immune responses between Group B1 and B2, we next assessed the TCR repertoire diversity across primary tumors and LNs in Groups A, B1, and B2 (Figure 3 and Supplementary Table S2). The Shannon diversity indices of tumor-infiltrating T cells showed no significant differences among the three groups for either TRA or TRB chains. In contrast, TCR diversity in the LNs was generally higher than in the tumors, consistent with the intrinsically diverse repertoire of lymphoid tissues. Especially, the Shannon indices of TRA and TRB in the LNs of Group B1 were lower than those of Group B2 and more closely resembled the diversity indices observed in the corresponding primary tumors. These findings suggest clonal skewing of T cell responses in the LNs of patients in Group B1, likely reflecting the expansion of tumor-specific clones and resulting in reduced TCR diversity.

### 2.5. Prognostic Discrimination Based on Immune Gene Expression in LNs

**Figure 4:**
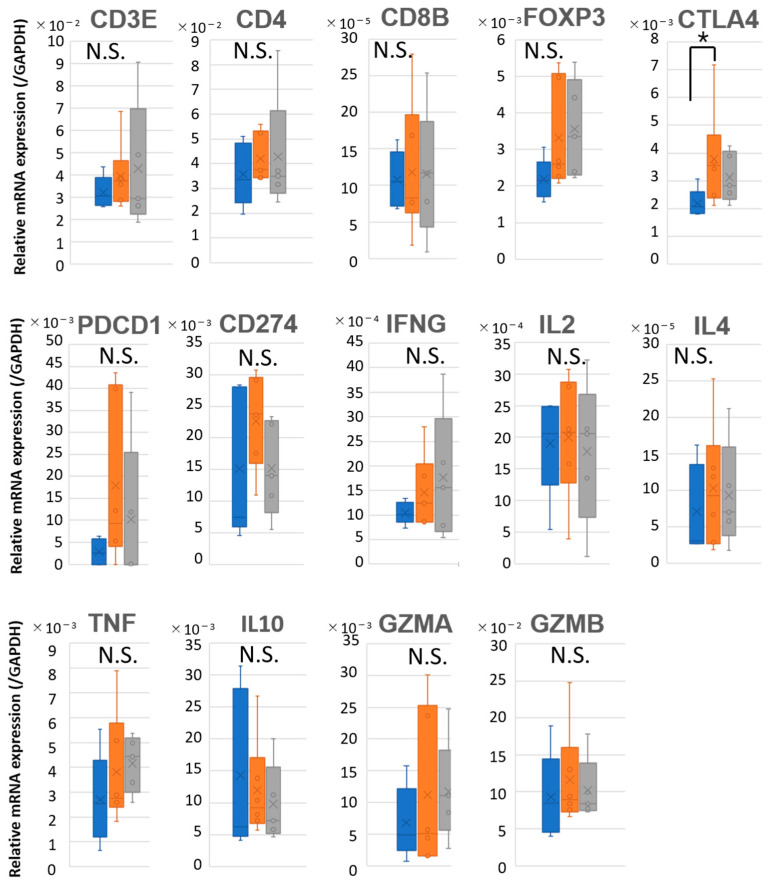
mRNA expression of immune-related genes in primary tumors from OSCC patients. Box plots showing the relative mRNA expression levels of T cell markers (*CD3E*, *CD4*, *CD8B*, *FOXP3*), immune checkpoint molecules (*CTLA4*, *PDCD1*, *CD274*), cytokines (*IFNG*, *IL2*, *IL4*, *TNF*, *IL10*), and cytotoxicity-related genes (*GZMA*, *GZMB*) in primary tumors of oral squamous cell carcinoma (OSCC) patients. mRNA expression levels were quantified by qPCR and normalized to *GAPDH* expression. Patients were categorized into three groups: Group A (no LN metastasis, blue), Group B1 (nodal metastasis without recurrence, orange), and Group B2 (nodal metastasis with recurrence, gray). Statistical comparisons were performed using the Kruskal–Wallis test. * *p* < 0.05; N.S., not significant.

To further evaluate whether the immune microenvironment within tumors and LNs could discriminate between patients with favorable (B1) and poor (B2) prognosis, we analyzed the expression of immune-related genes across the three groups (A, B1, and B2). Quantitative PCR (qPCR) was performed to assess the mRNA levels of T cell markers (*CD3E*, *CD4*, *CD8B*, *FOXP3*), exhaustion-associated genes (*PDCD1*, *CD274*, *CTLA4*), cytokines (*TNF*, *IFNG*, *IL2*, *IL4*, *IL10*), and cytotoxicity-related genes (*GZMA*, *GZMB*), with normalization to GAPDH. In the primary tumors, no significant differences in the expression of most immune-related genes were observed among the three groups. However, CTLA4 expression was relatively lower in Group A compared to Group B1 (Figure 4 and Supplementary Table S3).

**Figure 5:**
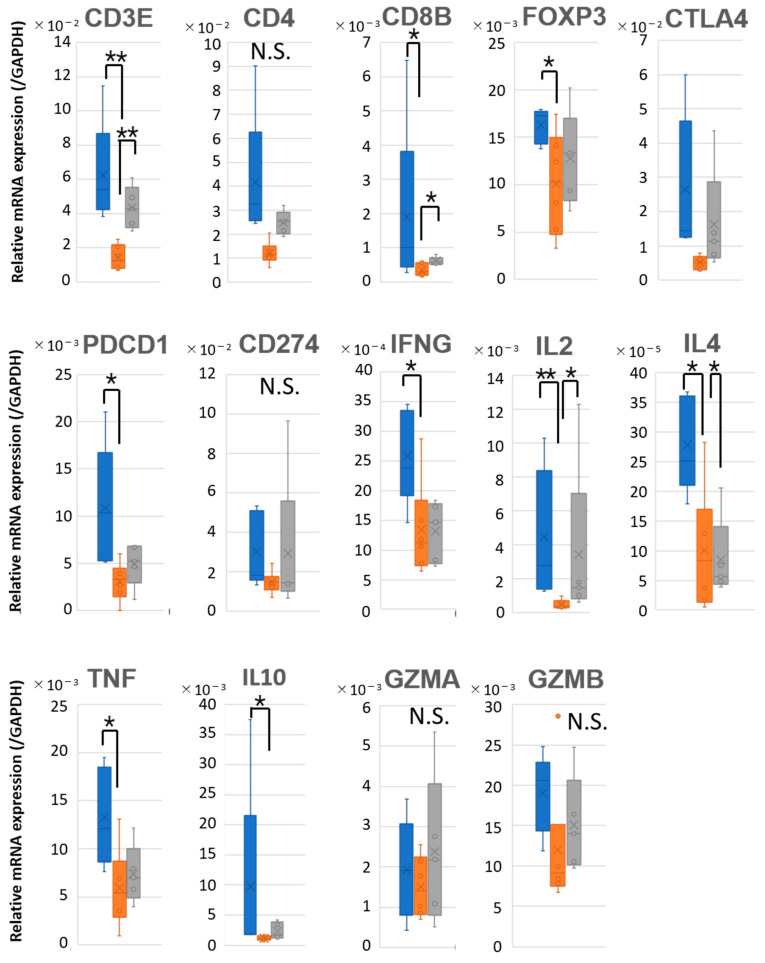
mRNA expression of immune-related genes in cervical LNs from OSCC patients. Box plots showing the relative mRNA expression levels of T cell markers (*CD3E*, *CD4*, *CD8B*, *FOXP3*), immune checkpoint molecules (*CTLA4*, *PDCD1*, *CD274*), cytokines (*IFNG*, *IL2*, *IL4*, *TNF*, *IL10*), and cytotoxicity-related genes (*GZMA*, *GZMB*) in cervical LNs of oral squamous cell carcinoma (OSCC) patients. mRNA expression levels were quantified by qPCR and normalized to *GAPDH* expression. Patients were categorized into Group A (no LN metastasis, blue), Group B1 (nodal metastasis without recurrence, orange), and Group B2 (nodal metastasis with recurrence, gray). Statistical comparisons were performed using the Kruskal–Wallis test. * *p* < 0.05; ** *p* < 0.01. N.S., not significant.

However, in LNs (Figure 5 and Supplementary Table S4), patients with nodal metastasis (Groups B1 and B2) showed generally lower expression levels of many immune-related genes compared to those without metastasis (Group A). When comparing the B1 and B2 groups, *CD3E*, *CD8B*, and *IL2* were expressed at lower levels in B1, whereas the expression of *IL4* was higher in B1 than in B2. These findings indicate that while gene expression profiles within tumors were insufficient to distinguish between B1 and B2 patients, the immune landscape in the LNs may offer potentially informative differences. In particular, the differential expression of helper cytokines such as *IL2* and *IL4*, in the absence of significant differences in effector molecules including *IFNG*, *TNF*, *GZMA*, and *GZMB*, may suggest potential differences in helper T cell activity in metastatic LNs, although the biological significance of these findings remains to be clarified. This highlights the importance of assessing nodal immune activity to better understand disease progression and prognosis.

## 3. Discussion

This study aimed to identify immune features that could distinguish between favorable and poor prognosis in OSCC patients with LN metastasis. By analyzing the T cell receptor (TCR) repertoire and immune-related gene expression in paired tumor and sentinel LN samples, we found that tumor-based analyses alone were insufficient to stratify the outcomes. In contrast, LN immune profiles—both in terms of TCR diversity and gene expression—successfully differentiated between patients with favorable prognosis (B1 group) and those who experienced recurrence (B2 group), despite both groups having histologically confirmed nodal metastasis.

LNs occupy a dual role in cancer biology: they are both sites of immune activation and conduits for metastatic dissemination [17]. Antigen-presenting cells (APCs) migrate from the tumor to draining LNs, where they present tumor-derived peptides to naïve T cells, triggering adaptive immune responses. Concurrently, tumor cells can exploit the lymphatic system to invade these same nodes, establishing metastatic niches. Several studies have suggested that immunologically “primed” LNs may paradoxically facilitate metastasis, potentially due to their active immune surveillance and antigen capture [7,8,9]. This dual nature supports our finding that LNs, rather than tumors, better reflect the dynamic interface between the immune system and cancer progression.

In our study, the TCR diversity within LNs of B1 patients was markedly reduced, with values approaching those observed in the corresponding primary tumors, suggesting the clonal expansion of tumor-specific T cells (Figure 3). While higher TCR diversity in tumor-free lymph nodes is generally associated with a healthy and adaptable immune repertoire, the immunological context in tumor-bearing lymph nodes differs fundamentally. In metastatic LNs, lower TCR diversity may reflect the clonal expansion of tumor-specific T cells actively responding to tumor antigens present within the node itself. This pattern likely represents an ongoing antitumor immune response rather than a failure of immune surveillance. Thus, in this setting, reduced diversity may be a surrogate for effective immunological engagement, consistent with favorable prognosis. This interpretation aligns with recent findings in tumor-infiltrated tissues in melanoma and lung cancer, where clonal T cell expansion is linked to improved outcomes [18,19]. In contrast, B2 patients exhibited higher TCR diversity, indicating a less focused or less activated T cell response within the LN. Gene expression analysis further supported these observations. While immune-related gene expression in primary tumors did not significantly differ among Groups A, B1, and B2 (Figure 4), clear differences were evident in the LNs (Figure 5). Both B1 and B2, which represent metastatic LNs, showed reduced immune gene expression compared to non-metastatic Group A. Compared to B2, B1 patients exhibited a significantly lower expression of T cell-associated markers and effector molecules such as *CD3E*, *CD8B*, and *IL2*, whereas *IL4* expression was paradoxically higher in B1, suggesting a skewed or compensatory helper T cell response. These findings imply that clonal expansion in B1 is not accompanied by broad effector activation and that differences in LN immune gene expression may reflect functionally distinct immune states. Although *PDCD1* (*PD-1*) and *CD274* (*PD-L1*) expression did not differ significantly between B1 and B2, the absence of upregulation does not exclude the possibility of T cell exhaustion. Therefore, the reduced effector profile observed in B1 may not be solely attributable to classical T cell exhaustion, although this interpretation is limited by a lack of functional assays to directly assess T cell activity or exhaustion.

These results suggest distinct immunological characteristics among the three groups of LNs. Group A represents immunologically intact, non-metastatic LNs with preserved structure and immune function. Group B1 comprises metastatic LNs undergoing the selective clonal expansion of T cells, yet with reduced diversity and suppressed expression of immune effector genes, indicating localized immune activation without broad responsiveness. In contrast, Group B2 also includes metastatic LNs but maintains relatively high TCR diversity and gene expression levels that more closely resemble those in non-metastatic nodes, suggesting a failure to mount an effective antitumor immune response. Taken together, these findings underscore the superior prognostic value of the immune profiling of LNs, particularly when combining TCR repertoire analysis and immune gene expression, compared to tumor-based analyses alone.

While ENE is widely recognized as a robust predictor of poor prognosis in OSCC, our findings showed some inconsistencies with this general trend. The presence of major ENE in several patients with favorable prognosis and its absence in others with recurrence suggests that ENE status alone may not fully capture the complexity of tumor behavior and host immune interactions. Possible explanations for this discrepancy include the relatively small sample size, retrospective design, and interobserver variability in pathological evaluation. Importantly, immune-related features—such as TCR diversity and gene expression in draining lymph nodes—may provide additional prognostic insight beyond traditional pathological markers. Future studies in larger, prospective cohorts are needed to clarify the relative contributions of ENE and immunological parameters.

Moreover, although we focused on T cell-related markers, it is important to consider the role of other immunoregulatory cells. Regulatory T cells, myeloid-derived suppressor cells (MDSCs), and immunosuppressive macrophages may contribute to the dysfunctional immune environment observed in B2 patients. Future studies should include spatial and single-cell analyses to evaluate the presence and localization of these populations within the LN microenvironment.

Another clinical implication of our findings may relate to whether adjuvant immunotherapy could be guided by the immune profiling of LN. Although speculative, it raises the question of whether patients with active immune responses (B1) might benefit from ICIs to sustain antitumor immunity or whether patients with immunologically quiescent LNs (B2) may require earlier intervention. Further mechanistic and interventional studies are necessary to test these hypotheses. In this context, understanding how LN immune status interacts with standard postoperative treatments, such as radiation and chemotherapy, will be critical for designing rational combination strategies.

There are several limitations to this study. First, it is a retrospective analysis with a limited cohort size (*n* = 16), which was further subdivided into three groups (A, B1, and B2). This small sample size inevitably limits statistical power and increases susceptibility to random variation, particularly when applying diversity metrics such as the Shannon index. Therefore, while the observed differences in TCR repertoire and immune gene expression are biologically plausible, they should be interpreted with caution due to the potential for sampling bias. Second, our approach relied on bulk TCR sequencing and qPCR-based gene expression, which does not capture the cellular or spatial heterogeneity of the immune landscape. Third, clinical variables such as tumor size, treatment modality, and comorbidities were not included in the immune stratification. Future prospective studies with larger patient cohorts and integrative analyses, including spatial transcriptomics and single-cell profiling, will be necessary to validate these findings and clarify how the LN immune microenvironment shapes clinical outcomes and therapeutic response in OSCC.

## 4. Materials and Methods

### 4.1. Sample Collection from OSCC Patients

The OSCC patients enrolled in this study are summarized based on their age, gender, tumor site, and TNM stage in Table 1. The primary tumor tissues and paired cervical LNs were collected from 16 OSCC patients who underwent surgical treatments at Tsurumi University Hospital and Kanto Rosai Hospital. Only patients with histologically confirmed OSCC, no prior neoadjuvant therapy, and HPV-negative status were included in this study. This study was approved by the Ethics Committees of Tsurumi University (No. 1608) and Kanto Rosai Hospital (Nos. 2018-025 and 2020-28). Written informed consent was obtained from all patients prior to the surgical treatment.

### 4.2. Histological Evaluation of OSCC Specimens

Oral squamous cell carcinoma (OSCC) specimens were immersed in 10% neutral buffered formalin, and paraffin blocks were prepared by sectioning the tissue at 4 µm thickness, followed by hematoxylin and eosin (H&E) staining. All resected tumor and lymph node specimens were processed similarly to ensure consistent histological evaluation. H&E staining was used to confirm the diagnosis of OSCC and to assess histopathological features, including tumor differentiation, invasion pattern, and the presence of lymph node metastasis. Histological evaluation of OSCC patients was classified according to the Yamamoto–Kohama (Y-K) classification in the primary tumors. The Y-K classification system is a widely used grading method in Japan for assessing the mode of invasion in oral cancers. It categorizes tumors from grade 1 (well-defined borders with minimal invasion) to grade 4D (diffuse infiltration with severe stromal involvement), reflecting the aggressiveness and infiltrative behavior of the tumor. This classification is useful in predicting local recurrence and prognosis.

The metastatic LNs, the type of extranodal extension, and mode of invasion were independently assessed by the board-certified pathologist. The final diagnosis was determined through joint review and consensus discussion. The mode of cervical LN metastases was classified based on their growth patterns within the LN. LNs where the metastatic focus was confined within the LN, with preserved follicular architecture, were designated as focal type. In contrast, LNs exhibiting widespread infiltration and proliferation of metastatic cells, leading to the complete replacement of the LN architecture, often with cyst formation or effacement of lymphoid follicles due to extensive invasion, were classified as cystic/invasive type (Supplementary Figure S1).

### 4.3. RNA Extraction

Fresh specimens of tumor tissue and paired submandibular LN specimens in OSCC patients were obtained during the operations and immediately soaked in RNAlater Stabilization Solution (Invitrogen, Waltham, MA, USA) and frozen at −80 °C until further processing. Total RNA was extracted from each specimen and purified with RNeasy Lipid Tissue Mini Kit (Qiagen, Hilden, Germany) according to the manufacturer’s instructions. RNA amounts and purity were measured with the Agilent 2100 bioanalyzer (Agilent Technologies, Palo Alto, CA, USA).

### 4.4. TCR CDR3 Region Sequencing and TCR Repertoire Analysis

TCR CDR3 regions were amplified and sequenced using the Repertoire Analysis Kit according to the manufacturer’s instructions (Repertoire Genesis, Ibaraki, Osaka, Japan). cDNA was synthesized using hTRA or hTRB-specific reverse transcription primers, which were subsequently conjugated with universal adapters. The first PCR was performed by unbiased PCR using a nested PCR primer set that amplified the V, J, and C regions of hTRA and the V, D, J, and C regions of hTRB. The first PCR products were confirmed by electrophoresis. The obtained PCR products were purified using Agencourt AMPure XP (Beckman Coulter, Brea, CA, USA). The concentration of the purified product was measured using a Qubit Fluorometer (Thermo Fisher, Waltham, MA, USA) and finally diluted to 4 nM. The final products were sequenced by Miseq (Illumina, San Diego, CA, USA). Raw sequencing reads were processed using the Repertoire Genesis analysis pipeline. Low-quality reads were filtered based on Phred quality scores, and sequences with ambiguous bases or short read lengths were excluded. Clonotypes were defined as unique combinations of V and J gene segments (for TRA) or V, D, and J gene segments (for TRB), with identical CDR3 amino acid sequences. Only productive (in-frame, stop codon-free) sequences were included in the analysis. The obtained sequence data were analyzed using TCR repertoire analysis software developed by Repertoire Genesis.

### 4.5. Quantitative Polymerase Chain Reaction

The expression levels of immune response-related genes, including T cell-related cluster of differentiation antigens and biomarkers, were measured by quantitative polymerase chain reaction (qPCR) using the Bio-Rad CFX96 system (Bio-Rad, Hercules, CA, USA). PCR primers for this study were purchased from Takara Bio Inc. (Shiga, Japan). The sequences of the primers are shown in Table 3.

cDNAs were synthesized from 500 ng of total RNA isolated from each specimen of OSCC using the Prime Script RT reagent Kit (TAKARA BIO INC., Shiga Japan) for each of the 10 qPCR reactions. The PCR mixture consisted of 5 μL of SsoFast EvaGreen Supermix (Bio-Rad, Hercules, CA, USA), 3.5 μL of RNase-free water, 0.5 μL of 5 μM primer mix, and 1 μL of cDNA, with a final total volume of 10 μL. This was then applied to the CFX96 system. The cycling conditions were as follows: 30 s at 95 °C followed by 50 cycles of 1 s at 95 °C and 5 s at 60 °C. At the end of each program, a melting curve analysis was performed from 70 °C to 94 °C to confirm the homogeneity of the PCR products. All assays were performed in triplicate and mean values were used to calculate gene expression levels. Gene expression levels were normalized to *GAPDH*, which was selected as the internal reference gene based on its consistent expression across all specimens in our preliminary evaluation. Other housekeeping genes were not assessed in this study, which may represent a limitation. Other housekeeping genes were not assessed in this study, which may represent a limitation. For calibration of the absolute quantification, a standard curve for each target was prepared by five 10-fold serial dilutions of the standard nucleotide that was generated by the linearized plasmid cloned with PCR products of each target. DNA sequencing (Sanger method) confirmed the sequence identity and was quantified by SmartSpec3000 (BIO-RAD, Hercules, CA, USA).

### 4.6. Statistical Analysis

As our data were non-parametric conditions, the Mann–Whitney U test and Kruskal–Wallis test were performed. All analyses were performed by SPSS version 24 (IBM, USA). A bilateral *p*-value below 0.05 was considered statistically significant.

## 5. Conclusions

Our data demonstrate that the immune profiling of LNs offers superior prognostic value over tumor analysis in OSCC patients with LN metastasis. The combined assessment of TCR repertoire diversity and immune gene expression in LN-based evaluation may aid in postoperative risk stratification and personalized postoperative management and could inform decisions regarding adjuvant therapy and follow-up strategies.

## Figures and Tables

**Table 1:** Patients Characteristics.

Patient	Age, Year	Gender	Primary Site	pTNM	Clinical Stage	Lymph Node Metastasis	Extranodal Extension	Local Recurrence	Distant Metastasis	Prognosis	PFS (Month)	OS (Month)	Group
1	74	F	Upper Gingiva	T2N0M0	2	—	—	—	—	Alive	72.3	72.3	A
2	79	M	Lower Gingiva	T4aN0M0	4A	—	—	—	—	Alive	70.8	70.8	A
3	71	M	Tongue, Esophageal	T2N0M0	2	—	—	—	—	Alive	70.1	70.1	A
4	61	F	Lower Gingiva	T3N0M0	3	—	—	—	—	Alive	65	65	A
5	64	M	Tongue	T3N0M0	3	—	—	—	—	Alive	61.1	61.1	A
6	71	F	Tongue	T1N1M0	3	+	—	—	—	Death from other causes	30.7	30.7	B1
7	64	M	Lower Gingiva	T4aN2bM0	4A	+	+	—	—	Alive	71.7	71.7	B1
8	67	M	Tongue	T2N2bM0	4A	+	—	—	—	Alive	71.4	71.4	B1
9	78	M	Upper Gingiva	T2N1M0	3	+	—	—	—	Alive	71.4	71.4	B1
10	75	M	Lower Gingiva	T1N1M0	3	+	—	—	—	Alive	67.8	67.8	B1
11	69	F	Tongue	T2N2bM0	3	+	—	—	—	Alive	64.1	64.1	B1
12	86	M	Lower Gingiva	T4N1M0	4A	+	—	+	—	Death	10.1	11.6	B2
13	79	F	Upper Gingiva	T4aN3bM0	4B	+	+	+	—	Death	1.6	18.1	B2
14	68	M	Tongue	T2N2cM0	4A	+	—	+	+	Death	3.1	11.2	B2
15	50	F	Tongue	T1N1M0	3	+	—	+	—	Death	14.6	35.6	B2
16	75	F	Tongue	T3N2bM0	4A	+	+	+	—	Death	5.4	14.3	B2

**Table 2:** Histological Characteristics in OSCC patients.

Patient	Age Year	Gender	Primary Site	pTNM	Clinical Stage	Histological Type of Primary Tumor (Y-K Classification)	Lymph Node Metastasis	Extranodal Extension	Type of Extranodal Extension ※	Histological Type of Metastatic Lymph Node	The Number of Metastatic Lymph Nodes	Group
1	74	F	Upper Gingiva	T2N0M0	2	3	—	—		—	0	A
2	79	M	Lower Gingiva	T4aN0M0	4A	4C	—	—		—	0	A
3	71	M	Tongue, Esophageal	T2N0M0	2	2	—	—		—	0	A
4	61	F	Lower Gingiva	T3N0M0	3	4C	—	—		—	0	A
5	64	M	Tongue	T3N0M0	3	2	—	—		—	0	A
6	71	F	Tongue	T1N1M0	3	1	+	—	No ENE Type 1	Focal	1	B1
7	64	M	Lower Gingiva	T4aN2bM0	4A	4C	+	+	Major ENE Type 4	Invasive/cystic	5	B1
8	67	M	Tongue	T2N2bM0	4A	3	+	—	No ENE Type 1	Focal	3	B1
9	78	M	Upper Gingiva	T2N1M0	3	4C	+	—	No ENE Type 1	Focal	1	B1
10	75	M	Lower Gingiva	T1N1M0	3	3	+	—	No ENE Type 2	Invasive/cystic	1	B1
11	69	F	Tongue	T2N2bM0	3	4C	+	—	No ENE Type 2	Invasive/cystic	1	B1
12	86	M	Lower Gingiva	T4N1M0	4A	4C	+	—	No ENE Type 1	Focal	2	B2
13	79	F	Upper Gingiva	T4aN3bM0	4B	4C	+	+	Major ENE Type 4	Invasive/cystic	1	B2
14	68	M	Tongue	T2N2cM0	4A	4D	+	—	No ENE Type 1	Focal	6	B2
15	50	F	Tongue	T1N1M0	3	4D	+	—	No ENE Type 1	Focal	1	B2
16	75	F	Tongue	T3N2bM0	4A	4C	+	+	Major ENE Type 4	Invasive/cystic	2	B2

※ Type of extranodal extension: Type 1. No ENE: tumor confined to the lymph node; Type 2. No ENE: tumor reaches and causes thickening of the capsule but is not through full thickness of capsule; Type 3. Minor ENE: tumor extends past capsule by 2 mm; Type 4. Major ENE: tumor extends past capsule by >2 mm; Type 5. Soft tissue metastasis: tumor mass without evidence of residual node or nodal architecture.

**Table 3:** The sequences of the primers.

*CD3E*	forward	TGCTGCTGCTGGTTTACTACTGG
	reverse	TCATAGTCTGGGTTGGGAACAGG
*CD4*	forward	AAGTGAACCTGGTGGTGATGAGA
	reverse	CTCCCGCTTCGAGACCTTTG
*CD8B*	forward	GCCGGAAGACAGTGGCATCT
	reverse	TCTCTTCTTGAGGGTGGACTTCTTG
*FOXP3*	forward	GGGTAGCCATGGAAACAGCA
	reverse	TCGCATGTTGTGGAACTTGAAGTA
*CTLA4*	forward	ATCTGCAAGGTGGAGCTCATGTA
	reverse	ATCTGGGCACGGTTCTGGA
*PDCD1*	forward	GGTGCCGACTACAAGCGAATTAC
	reverse	GGAATTGGTGGTGGTGGTCTTAC
*CD274*	forward	AAATGGAACCTGGCGAAAGC
	reverse	GATGAGCCCCTCAGGCATTT
*IFNG*	forward	CTTTAAAGATGACCAGAGCATCCAA
	reverse	GGCGACAGTTCAGCCATCAC
*IL2*	forward	AGACCCAGGGACTTAATCAGCAATA
	reverse	TTCTACAATGGTTGCTGTCTCATCA
*IL4*	forward	GACTCGCCTACAAAGCCCAGA
	reverse	AGCTGCTTGTGCCTGTGGAA
*TNF*	forward	GAATGAGCTTCTGGAGGCTTG
	reverse	TGGAGTTGATGTCAGTCACTTGG
*IL10*	forward	GGCCCAATTGACTGACAGGA
	reverse	TCAAACTCACTCATGGCTTTGTAGA
*GZMA*	forward	GACTGGGTGTTGACTGCAGCTC
	reverse	TGGCTGGGTCATAGCATGG
*GZMB*	forward	TGACAGCTGCTCACTGTTGG
	reverse	GTTCTTAGGATTATAGGCTGGATGG
*GAPDH*	forward	GCACCGTCAAGGCTGAGAAC
	reverse	TGGTGAAGACGCCAGTGGA

## Data Availability

The original contributions presented in this study are included in the article/Supplementary Materials. Further inquiries can be directed to the corresponding author.

## Supplementary Materials

The following supporting information can be downloaded at: https://www.mdpi.com/article/10.3390/ijms26157073/s1.

## Abbreviations

The following abbreviations are used in this manuscript:

TDLN	Tumor-draining lymph node
OSCC	Oral squamous cell carcinoma
LN	Lymph node
TCR	T cell receptor
CDR3	Complementarity-determining region 3
qPCR	quantitative polymerase chain reaction
GAPDH	Glyceraldehyde-3-Phosphate Dehydrogenase
CDLN	Cervical draining lymph node
APC	Antigen-presenting cell
PFS	Progression-free survival
OS	Overall survival
Y-K	Yamamoto–Kohama
ENE	Extranodal extension
TRA	T cell receptor alpha chain
TRB	T cell receptor beta chain
FOXP3	Forkhead box P3
CTLA4	Cytotoxic T-lymphocyte associated protein 4
GZM	Granzyme
PDCD1	Programmed cell death protein 1 (PD-1)
CD274	Cluster of differentiation 274, programmed death-ligand 1 (PD-L1)
Treg	Regulatory T cell
MDSC	Myeloid-derived suppressor cell
ICI	Immune checkpoint inhibitor
