# Supplementary material for: Prognostic Implications of T Cell Receptor Repertoire Diversity in Cervical Lymph Nodes of Oral Squamous Cell Carcinoma Patients
Source: Int J Mol Sci. 2025 Jul 23;26(15):7073. doi: 10.3390/ijms26157073 (PMC12346881; doi:10.3390/ijms26157073)
Supplement: Supplementary file 1 [file ijms-26-07073-s001.zip › Spplementary Figure 1.pdf]

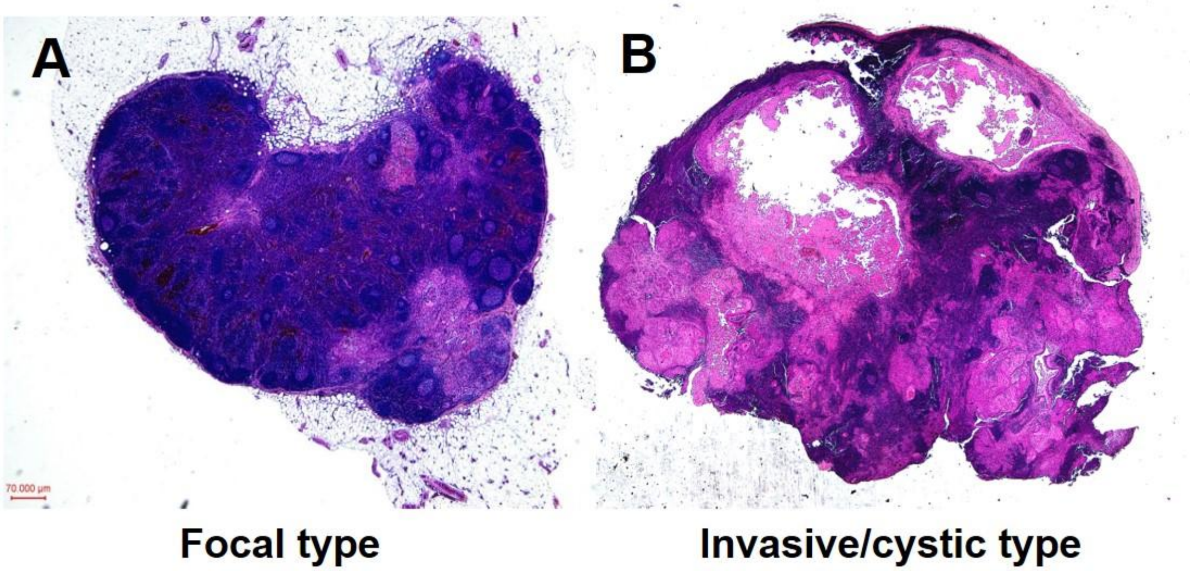


**Supplementary Figure 1.**
Representative histological features of cervical lymph node metastases. Focal type shows confined metastatic foci with preserved follicular architecture. Cystic/invasive type exhibits widespread infiltration, architectural effacement, and cyst formation.
